# A Closer Look at the Validity and Reliability of the Persian Versions of National Institute of Health Stroke Scale and Modified National Institute of Health Stroke Scale in Hospitalized Patients

**DOI:** 10.31661/gmj.v8i0.1587

**Published:** 2019-08-19

**Authors:** Mehrdad Amir Behghadami, Ali Janati

**Affiliations:** ^1^Iranian Center of Excellence in Health Management (IceHM), Department of Health Service Management, School of Management and Medical Informatics, Tabriz University of Medical Sciences, Tabriz, Iran; ^2^Student Research Committee (SRC), Tabriz University of Medical Sciences, Tabriz, Iran

**Keywords:** Validity, Reliability, Psychometric Properties

## Dear Editor,

The present letter concerns the article written by Dehghani et al. [1]. First, we appreciate the efforts made by the editors of Galen Medical Journal to help publish such an important article. However, the present methodological approach of the mentioned study indicates some flaws resulting from the negligence of the authors, which has led to an ambiguous interpretation of the results. This letter aims to help readers understand the matter better. Therefore, some of the points expressed in this letter indicate what is yet essential to confirm valid and reliable scales. Psychometric studies can be very effective and valuable for healthcare workers since such studies provide valid and reliable scales [2]. One the one hand, an accurate and appropriate study design, helps researchers plan the study decently. On the other hand, it can direct readers either toward what has been or will be conducted in a study. Hence, to be more transparent, it is suggested that in psychometric studies, researchers apply an appropriate study design. In recent years, the increase in the number of multicultural studies has urged the need to adapt scales to be used in other languages [3]. Hence, depending on different cultures, the scales should be culturally modified and adapted [2]. Regarding this point, cross-cultural adaptation should be used as the study design. This design, i.e., cross-cultural adaptation, consists of translation, adaptation, calculation of validity, reliability, and, responsiveness [3]. Nevertheless, it seems that the validity assessment needs to be clarified. Content validity is a crucial component of psychometric studies, which must be performed independent of the translation phase [4]. The content validity of scales can be assessed using modified Kapaa (modified content validity index [CVI]), which employs both quantitative and qualitative approach. It is done in a way that Persian version of the scales is assessed through using the viewpoints of the panel of experts [5]. This panel consists of specialists who have research experience or worked in the field [5]. And, the specialists are asked to present their own ideas to improve the quality of the scales and also to judge the existing items in terms of clarity and relevance [6]. These two criteria can be separately considered on a 4-point Likert scale by the specialists [6, 7]. As a result, to calculate the Kappa coefficient (modified CVI) based on these two criteria, each item in the Likert scale is ranked according to experts’ view [8]. Given this, for each item of the scale, Kappa (modified CVI) is calculated as the number of experts, who ranked 3 or 4, divided by the total number of the experts [9]. In conclusion, as psychometric studies present valid and reliable scales to investigate health-related issues and design future studies, it is crucial that the results should be reported in an accurate method. To improve validity, it is suggested that the authors report their findings on the content validity of the scales so that the CVI of each item on the scales is determined.

## Conflict of Interest

Not to declare.

## References

[1] Dehghani R, Borhanihaghighi A, Shariat A, Nami M, Nazeri M, Foroughi AA (2019). Validity and reliability of the persian versions of national institute of health stroke scale and modified national institute of health stroke scale in hospitalized patients. Galen.

[2] Yasir AS (2016). Cross Cultural Adaptation & Psychometric Validation of Instruments: Step-wise. International journal of psychiatry.

[3] Arafat SY, Chowdhury HR, Qusar M, Hafez M (2016). Cross cultural adaptation & psychometric validation of research instruments: A methodological review. Behavioral Health.

[4] Taghizadeh Z, Ebadi A, Montazeri A, Shahvari Z, Tavousi M, Bagherzadeh R (2017). Psychometric properties of health related measures Part 1: Translation, development, and content and face validity. Payesh Health Monit.

[5] Davis LL (1992). Instrument review: Getting the most from a panel of experts. Applied nursing research.

[6] Polit DF, Beck CT, Owen SV (2007). Is the CVI an acceptable indicator of content validity? Appraisal and recommendations. Res Nurs Health.

[7] Haynes SN, Richard D, Kubany ES (1995). Content validity in psychological assessment: A functional approach to concepts and methods. Psychol Assess.

[8] Amir Behghadami M, Janati A (2019). A second look at the reliability and validity of the Persian language version of the female lower urinary tract symptoms’ long form questionnaire. Iranian J Nursing Midwifery Res.

[9] Amir Behghadami M, Janati A, Sadeghi-Bazargani H (2019). Developing and validating an instrument to assess non-hospital health centers’ preparedness to provide initial emergency care. a study protocol BMJ Open.

